# Graphene Flakes for Electronic Applications: DC Plasma Jet-Assisted Synthesis

**DOI:** 10.3390/nano10102050

**Published:** 2020-10-16

**Authors:** Irina V. Antonova, Marina B. Shavelkina, Artem I. Ivanov, Regina A. Soots, Peter P. Ivanov, Alexey N. Bocharov

**Affiliations:** 1 Rzhanov Institute of Semiconductor Physics SB RAS, Lavrentieva 13, Novosibirsk 630090, Russia; aivanov@isp.nsc.ru (A.I.I.); soots@isp.nsc.ru (R.A.S.); 2 Department of Semiconductor Devices and Microelectronics, Novosibirsk State Technical University, R Marx str. 20, Novosibirsk 630073, Russia; 3 Joint Institute for High Temperatures RAS, Izhorskaya st. 13 Bd.2, Moscow 125412, Russia; mshavelkina@gmail.com (M.B.S.); peter-p-ivanov@yandex.ru (P.P.I.); bocharov@ihed.ras.ru (A.N.B.)

**Keywords:** DC plasma synthesis, graphene flakes, quasi-one-dimensional flow, composite films, electrical properties, 2D printing technologies

## Abstract

The possibility of graphene synthesis (the bottom-up approach) in plasma and the effective control of the morphology and electrical properties of graphene-based layers were demonstrated. Graphene flakes were grown in a plasma jet generated by a direct current plasma torch with helium and argon as the plasma-forming gases. In the case of argon plasma, the synthesized graphene flakes were relatively thick (2–6 nm) and non-conductive. In helium plasma, for the first time, graphene with a predominance of monolayer flakes and high conductivity was grown in a significant amount using an industrial plasma torch. One-dimensional (1D) flow modeling shows that the helium plasma is a less charged environment providing the formation of thinner graphene flakes with low defect density. These flakes might be used for a water-based suspension of the graphene with PEDOT:PSS (poly(3,4-ethylenedioxythiophene): polystyrene sulfonate) composite to create the structures employing the 2D printing technologies. Good structural quality, low layer resistance, and good mechanical strength combined with the ability to obtain a large amount of the graphene powder, and to control the parameters of the synthesized particles make this material promising for various applications and, above all, for sensors and other devices for flexible electronics and the Internet of things ecosystem.

## 1. Introduction

Graphene, due to its unique electronic characteristics [1,2], is one of the in-demand materials for various applications in electronics: in the design and manufacture of manifold functional electronic [3,4], biological and bioelectronic medical [5] and non-medical [6] devices, for printed and flexible electronics [7,8], and in the development of materials and composites with controlled properties [9,10]. As a rule, to obtain the graphene fragments, various methods of graphite processing are used: thermal expanded graphite stratification [11], use of a dispersants [12], electrochemical stratification [13], etc. The main problem with the existing methods is the difficulty of obtaining the monolayer nanoparticles. Their thickness is usually several nanometers, which is a significant limitation, in terms of maximum flexibility and the required composition (volume of graphene), in electronic applications [14], in printing technologies [15], as well as for application in composites [16].

Unlike the ideal graphene (a monolayer of the sp^2^-carbon atoms) the material for the printed electronics should have, in addition to good electronic characteristics (high conductivity and carrier mobility), (a) proven and scalable technology for producing graphene flakes; (b) a low cost of starting materials and manufacturing of the graphene suspension and ink; (c) high physical and mechanical stability of the starting materials and devices under the operating conditions; (d) formation, after the solvent evaporation, of the entire printed layer with good adhesion and interaction between the particles. Compliance of most of these properties is conditioned by the production of thin (preferably monolayer) flakes. However, the graphite exfoliation does not, as a rule, satisfy this important requirement. The way out of this contradiction is in changing the approach: in the transition from graphite exfoliation to the synthesis of the graphene flakes.

Graphene might be synthesized in two ways: the bottom-up and the top-down. The top-down method of graphene production is based on the exfoliation of thin layers from bulk graphite including a mechanical cleavage using Scotch tape, direct liquid phase exfoliation of graphene, exfoliation of a graphite intercalation compound with the help of ultrasonication, or oxidation of graphite to graphene oxide (GO) [17,18,19]. Graphene produced from graphite by direct exfoliation methods has relatively high crystal quality (high electrical conductivity, less crystal defect) but the production yield is still so low that is not enough for any practical application [20]. Liquid phase exfoliation of graphene is a widely used method due to its multiple advantages in terms of high performance, low cost, and technological solutions; however, the graphene does not have high quality and flakes have a thickness of few nanometers (typically 2–6 nm) [12]. Graphene produced through the oxidation of graphite to GO, exfoliation of resulting GO, and reduction of exfoliated graphene oxide (RGO) is the most popular method (Hummer’s method) [21,22]. This method often yields few-layer graphene flakes which have a thickness of up to 2 nm and lateral length in the range from several hundred nanometers to several micrometers. This method is suitable for mass production of RGO flakes with relatively low production cost and can yield large quantities of graphene flakes for a variety of applications, where high purity is not required such as fillers for polymer nanocomposites, coating, conductive ink paste, energy storage applications (electrode for lithium-ion, lithium sulfide, lithium-air batteries, and supercapacitors), etc. [21,22,23]. The bottom-up approach is based on the growth of carbon atoms into high-quality two-dimensional carbon layers using chemical vapor deposition (CVD) or epitaxial growth on SiC, where well-controlled thickness (number of layers) can be undertaken using different substrate catalysts and growing parameters [24,25]. By the CVD method large-area, single and few-layer graphene poly- or monocrystalline films are grown. CVD graphene has a large area, high quality, and this method has the best potential for mass production of high-purity graphene. The practical use of this approach is limited due to its high cost, complexity in the transfer process, and the difficulty to scale-up. Therefore, simple, cheap approaches for the creation of graphene flakes are required for a wide spectrum of applications. Modern graphene printing methods are based on solutions of the liquid-phase ink consisting of graphene (or its derivatives) fillers in stabilized solutions [26]: therefore, the material should be without undesirable additives.

The authors of [27] show that with the application of plasma, the small-layer flakes are formed without using substrates, and the synthesis process itself is fast and controlled [28]. A feature of the plasma synthesis is as follows: when there are cheap gaseous sources of carbon or alcohols, the carbon particle fragments are formed in the gas phase of the plasma stream [29,30]. The synthesized flakes might contain from 1 to 10 and more layers with the different defect density depending on the conditions. Thus, the use of such technologies will significantly reduce the final cost of electronic devices. Application of the direct current (DC) arc torch plasma jets provides the advantages of the property controllability. The experimental and the theoretical studies of the graphene synthesis process show that it is possible to control not only the geometry (thickness, lateral size) of the graphene flakes but also their shape varying the carbon-to-hydrogen ratio in the gas phase [30]. We apply the carbon nanostructures synthesized using this approach to fabricate the supercapacitor electrodes [31].

The present work is aimed at searching for the conditions for efficient bottom-up synthesis of the graphene flakes utilizing the DC plasma torch as well as at investigation of the structural and the electrical properties of the conducting layers produced via the printing technologies. As a result, we show that the graphene powder with a predominance of monolayer flakes might be successfully applied in the 2D printing technologies and, as an example, in the production of the conductive layers for a wide spectrum of applications: various sensors and other devices of flexible electronics.

## 2. Materials and Methods

To synthesize the graphene, we applied the plasma-chemical approach based on the use of a 40 kW DC plasma torch with the expanding channel of the output electrode and the vortex stabilization of the plasma jet. The design feature of the plasma torch (expanding channel of the output electrode) and the tangential input make it possible to obtain stable plasma jets within a wide range of parametric studies [31]. A more detailed description of the installation is given in [31,32]. The task of the present experiment was to synthesize the small-size monolayer carbon nanoparticles. Therefore, in the experiment, we took the most famous inert gases: helium and argon, with the pressure range below atmospheric: from 150 to 500 Torr. At the atmospheric pressure, the plasma jets are less stable and the electrode material is detected in the synthesis products. We took a propane-butane mixture, 65:35 mass%, as the source of carbon, with the mass flow rate of 0.1 g/s. Hereafter, we use the C-Ar and the C-He notations for the flakes obtained in Ar and He plasmas, respectively. For the C-He samples, the helium flow rate was 0.75 g/s; for the C-Ar samples, the argon flow rate was 3.75 g/s. The product of the plasma-chemical synthesis is a powder (the so-called dry powder) with the typical bulk density of 0.0001 g/cm^3^.

To study morphology and the local properties with high spatial resolution, we employed electron microscopy and Raman spectroscopy. We used the Hitachi S5500 scanning electron microscope (Hitachi High-Technologies Corp (Microscopy), Japan) with in-lens technology (STEM mode) and a Nova NanoSem 650 raster electron microscope (Nova NanoSem™, FEI Co., Hillsboro, OR, USA) with the standard processing technique (SEM mode). We recorded the Raman spectra at room temperature on a INTEGRA Spectra spectrometer (INTEGRA Spectra, NT-MDT, Moscow, Russia) using λ = 532 nm exciting radiation. We applied X-ray element microanalysis (EDAX) (Nova NanoSem 650, FEI Co., Hillsboro, OR, USA) to identify the chemical elements and to determine their quantitative content as well as the dynamic light scattering (DLS) method) (Anton Paar GmbH, Graz, Austria) to measure the nanoparticle size. The DLS method makes it also possible to determine the diffusion coefficient of the dispersed particles in the liquid using analysis of the correlation function of the scattered light-intensity fluctuations. Then, from the diffusion coefficient, we calculated the nanoparticle radii. We employed a Solver PRO NT-MDT (NT-MDT, Moscow, Russia) scanning microscope to obtain atomic force microscopy (AFM) images from the surface of the examined films and substrates and to evaluate the sample thicknesses. The measurements were carried out in contact and semi-contact modes. A X-ray photoelectron spectroscopy (XPS) measurements were carried out in an ultrahigh-vacuum chamber equipped with an X-ray source (Mg Kα, 12.5 kV, 250 W) and a hemispherical energy analyzer Phoibos (150 SPECS Gmb, Berlin, Germany).

We printed the films on DMP-2831 Dimatix FUJIFILM jet printer (Fujifilm, Lebanon, PA, USA). After printing a layer, the film was subjected to a drying procedure at 60 °C to exclude the possible mixing effects. Then we studied the layer resistance of the fabricated films using a four-probe JANDEL equipment and the HM21 Test Unit at room temperature (Jandel Engineering Limited, Limslade, UK). To measure the current-voltage (I-V) characteristics of the fabricated structures, we employed a high-precision Keithley picoampere-meter (model 6485, Keithley Instruments, Cleveland, OH, USA) at room temperature, with Ag alloy contacts.

When studying the cyclic deformations, we performed simultaneous testing of the electrical parameters of the material or structures utilizing the home-made installations applying tensile and compressive loads to the tested films. The strain was estimated using the well-known equation ε = (d + t)/2r where d is the flexible substrate thickness (d = 100 mm), t is the film thickness, and r is the substrate bending radius. The film thickness t < 500 nm is negligible as compared to that of the substrate.

## 3. Experimental Results

### 3.1. Structure Properties of the Graphene Flakes

**Figure 1:**
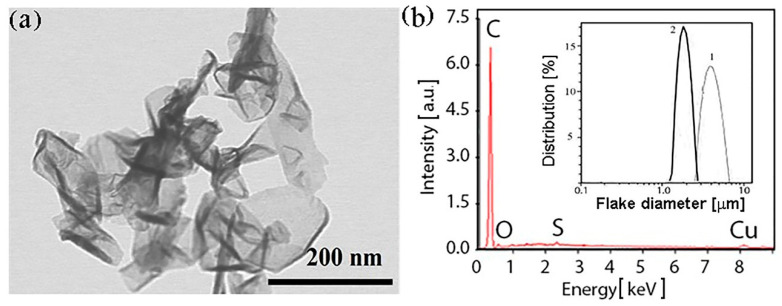
(**a**) The scanning electron microscope with in-lens technology (STEM) image of morphology of the graphene flakes synthesized in helium plasma; (**b**) Carbon content in the synthesized graphene determined by the X-ray element microanalysis (EDAX) was the same for C-Ar and C-He flakes. The insert: particle distribution over the size in the liquid ethanol medium measured by the dynamic light scattering (DLS) method: (*1*) C-Ar, (*2*) C-He.

The EDAX results for both types of sample (see Figure 1b) show that the carbon portion equals 96.7 atomic% (95.5 weight%) and does not differ for the C-Ar and the C-He particles. The oxygen fraction does not exceed 3.1 atomic%; thus, we claim that the synthesized particles are precisely graphene (multigraphene). The copper with sulfur additive observed in Figure 1b is material of the arc plasma torch electrodes (electrode erosion occurs during plasma synthesis), and the EDAX method is quite sensitive for trapping an admixture of the electrode material. The particle distribution over the size in the ethanol liquid medium before disintegration (see the inset in Figure 1b) shows a maximum of 4 μm for the C-Ar (curve 1) and of 2 μm for the C-He (curve 2) flakes. During further flake processing, their sizes decreased and are given in Table 1.

The specific surface area for both types of flakes measured by the standard Brunauer–Emmett–Teller (BET) method was ~500 m^2^/g. For comparison, [33] presents the theoretical estimate: the specific surface for graphene should be 2600 m^2^/g.

Figure 1 demonstrates the morphology of the sample synthesized in helium. Note that the sample consists of flakes with different thickness, their maximal thickness reaches ~2 nm, the number of layers is 1–5, the lateral size is 50–200 nm.

**Figure 2:**
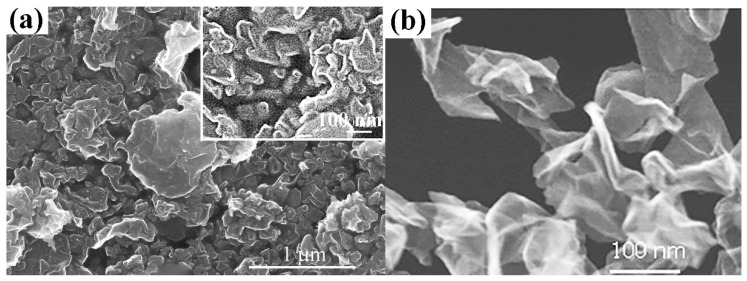
Scanning electron microscope (SEM) images of the morphology of the graphene flakes synthesized in the plasma of (**a**) argon in different scales and (**b**) helium.

Figure 2 shows the STEM images of the films consisting of the graphene particles synthesized in helium and argon. Figure 2a shows that the morphology of the flakes synthesized in argon plasma is complicated, mainly consisting of the carbon particle dendrites with a size of 50–100 nm. On their background, the flakes with the lateral size 300–800 nm are seen. Unlike the samples synthesized in argon, the C-He flakes are homogeneous thin flakes with a lateral size of up to 200 nm (Figure 2b). Moreover, when comparing the C-Ar and the C-He images, note that the C-He flakes look finer than the C-Ar ones. Table 1 presents the typical thickness and size of both types of flake, according to the SEM and the AFM data. For comparison, it also presents the typical parameters of the flakes, hereafter, the C-M obtained from the natural purified graphite using mechanical exfoliation employing a laboratory dispersant. The comparison shows that the use of helium plasma makes it possible to obtain the finest flakes. This is the most important parameter from the standpoint of the printing technologies and applications of the films obtained.

**Figure 3:**
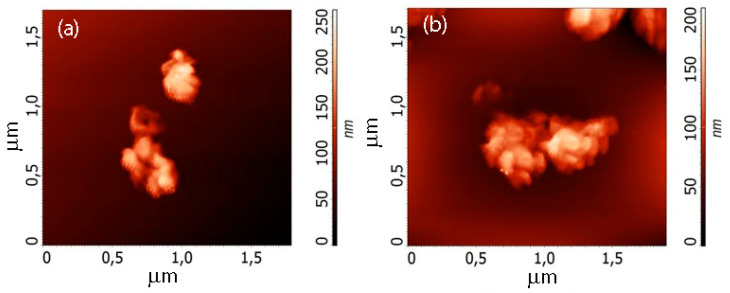
Atomic force microscopy (AFM) images of the suspension particle clusters: (**a**) C-Ar, (**b**) C-He.

To prepare the suspensions, we mixed 10 mg of the dry graphene flakes powder with 50 mL of the 0.7:0.3 ethyl alcohol-water solution, and added the surfactant (Trilon B, 0.025 mL, 0.05%). After sonication and centrifugation aimed at separation into individual flakes, we deposited the suspension on the silicon surface. Figure 3 shows the AFM images of the flakes. The lateral sizes and especially the thickness of flakes created in helium plasma are smaller than those parameters of the C-Ar flakes. The flakes are seen to be clustered. For this reason, we repeated sonication and centrifugation several times. Then, to remove the excess surfactant, we replaced the above solution with pure alcohol four times. Filtration made it possible to isolate the most interesting part with sizes less than 400 nm. After obtaining the homogeneous suspension, we deposited its droplets on the SiO_2_/Si substrate.

**Figure 4:**
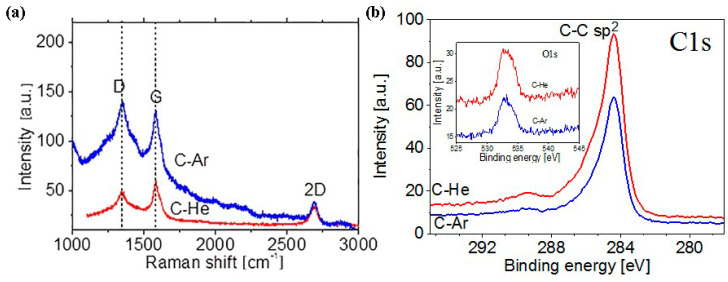
(**a**) Raman spectra were measured for the films obtained from the C-Ar and the C-He suspensions. (**b**) X-ray photoelectron spectroscopy (XPS) spectra for C-Ar and C-He films.

Figure 4 shows the Raman spectra of the C-Ar and the C-He films; the spectra contain the standard peak set typical for graphene or multigraphene: D (1350 cm^−1^), G (1585 cm^−1^) 2D (2700 cm^−1^). Note that the D-line structure for the C-Ar film attests to the fact that the synthesized flakes contain not one but several layers [34,35]. The C-He film is formed by thinner flakes since the D-line does not have distinct wings (as does that line for the C-Ar film). The ratio of line intensities, I_D_/I_G_ > 1 for the C-Ar film indicates a significantly higher defect concentration than the C-He film where I_D_/I_G_ < 1.

### 3.2. Electrical Properties of the Layers

The C-He film created by the drops turned out to be loose and crumbled. The C-Ar suspension made it possible to form denser and truly solid film. The thickness of the films was large, of the order of a micron. After drying, we measured the layer resistance of the films using the four-probe head. The values obtained were 1.3 MΩ/sq and 5.0–5.6 MΩ/sq for the C-Ar and the C-He suspensions, respectively. Our attempt to consolidate the film by a mechanical action did not lead to a change in the C-Ar film resistance and destroyed the C-He film. Thus, in the C-He film flakes do not form a unified material due to the relatively high concentration of surfactants on the surface of the fine flakes; that very fact was the reason for the high resistance. In contrast, the C-Ar film was formed as a singular layer and its high resistance is most likely related to the high resistance of the particles themselves.

Crystal phase of the obtained materials were determined by XPS. XPS spectra for C-Ar and C-He samples are given in Figure 4b. In the XPS spectra one can see a narrow intense peak with a maximum near 284.5 eV, which is characteristic of the C–C bond energy in sp^2^ hybridization. Also the C1s spectra of the both graphene flakes includes peaks centered at about 286.4, 287, and 289 eV, corresponding to C–O, –C=O and –COO^−^ groups, respectively [36,37,38]. The presence of weak peaks associated with oxygen-containing groups can be associated with the adsorption of oxygen on the surface of the flakes. There is practically no difference between the spectra for the C-Ar and C-He samples.

To improve the conditions for the C-He film formation from the individual particles, we added to the above-described suspension a 1% PEDOT:PSS (poly(3,4-ethylenedioxythiophene): polystyrene sulfonate composite) water solution, the total composition being 2:1 or 0.05 weight% in the film. This led to radical changes in the formed film quality. Here, for the C-He film, we obtained the layer resistance of 600–800 Ω/sq (for the film of submicron thickness) and 1.4–2.6 kΩ/sq for the thin (about 200 nm) film. For comparison, a thick PEDOT:PSS film (more than a micron) had the resistance of 3.6–4.2 kΩ/sq, and the thinner films (~200 nm) with the resistance of ~10 kΩ/sq. For the composite film with the C-Ar suspension, the resistance coincided with that of the PEDOT:PSS, that is, the contribution of the C-Ar particles was not noticeable.

We performed the Hall measurements (0.42 Tesla, the current of 500 μA) for the C-He based composite film, its thickness being about 5 μm, the sheet concentration was *n* = 3.1 × 10^15^ cm^–2^: it corresponds to the volume plane of ~7 × 10^18^ cm^–3^. For a thinner film, *n* = 3 × 10^13^ cm^–2^. The carrier mobility in the films turned to be relatively high: μ = 6–90 cm^2^/Vs. The carrier mobility values of ~90 cm^2^/*V*s are usually obtained for the 1–2 monolayer thick graphene particle films [3,12].

### 3.3. Modeling of Properties of Helium and Argon Plasma Jets

Numerical simulation of the plasma flow was performed using the quasi-one-dimensional approach when the two-dimensional system of equations was transformed into the one-dimensional system employing integration along with one of two coordinates under the given profiles of velocity and temperature [22]. Plasma flow is strongly dependent on the properties of the working medium. Here, the thermodynamic and transport properties were determined assuming the local thermodynamic equilibrium for the heterogeneous mixture of chemically reacting components. The method of sequential equilibration of reactions using the minimization of Gibbs potential was used. Properties of individual substances were from the IVTANTHERMO database [39].

**Figure 5:**
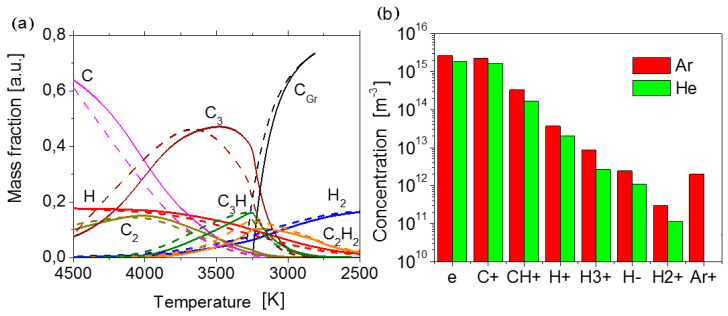
(**a**) Evolution of the composition of plasma jets during the cooling: solid lines correspond to helium plasma, and dashed line to argon plasma. (**b**) The concentration of charged particles and electrons (e) under the temperature of the beginning of the carbon condensation (3304 K for Ar and 3270 K for He).

As a result, temperature profiles along the jet axis and corresponding equilibrium plasma compositions were calculated. Figure 5a illustrates the evolution of the composition of helium plasma with the addition of the feedstock along the temperature axis during the cooling. Mass fractions are related to the feedstock mass flow rate. In the temperature range, where a drastic change of composition occurs, atomic hydrogen turns into the molecular hydrogen. Atomic carbon at the first step forms precursors of solid carbon like C_2_, C_3_, C_3_H, C_2_H_2_, and later at the temperature 3270 K the condensation of carbon into graphene/graphite (C_Gr_) commences. Similar curves for the case of argon plasma are also given in Figure 5a for comparison. In the case of argon plasma, chemical reactions go on under the higher temperature (especially remarkable are the curves C, C_3_, and C_gr_). The condensation begins a little bit earlier at 3304 K. It means a longer sojourn of particles under high temperatures. Figure 5b reveals the difference of composition in two considered cases under the temperature of the beginning of the condensation of carbon. As one can see, the helium plasma is a less charged environment in comparison with argon plasma. Lower concentration of charged components provides the formation of thinner graphene flakes with low defect density. Thus, we have to expect better structural and electrical properties for graphene flakes synthesized in helium plasma. This conclusion is correlated well with the experimental results obtained.

### 3.4. Two-Dimensional (2D) Printed Structures

**Figure 6:**
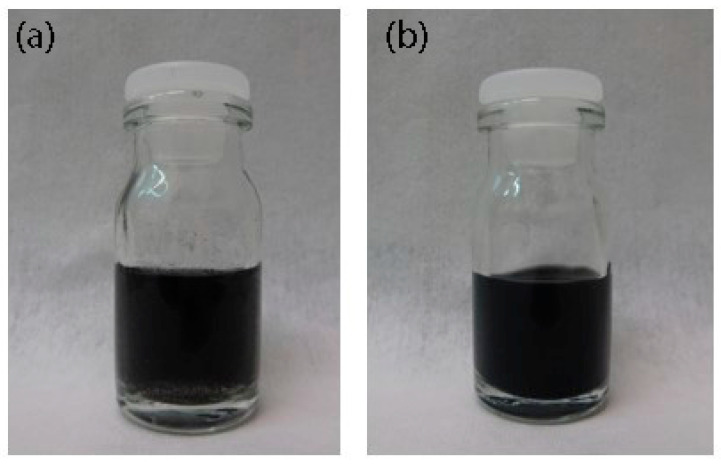
Ink photos after (**a**) preparation and (**b**) eight months later.

To print on the 2D printer, we prepared the ink from the C-He: PEDOT:PSS conductive composite suspension. Figure 6 illustrates the resulting long-term ink stability. The reason for that high stability is in thin suspension flakes, with a large number of monolayer flakes.

**Figure 7:**
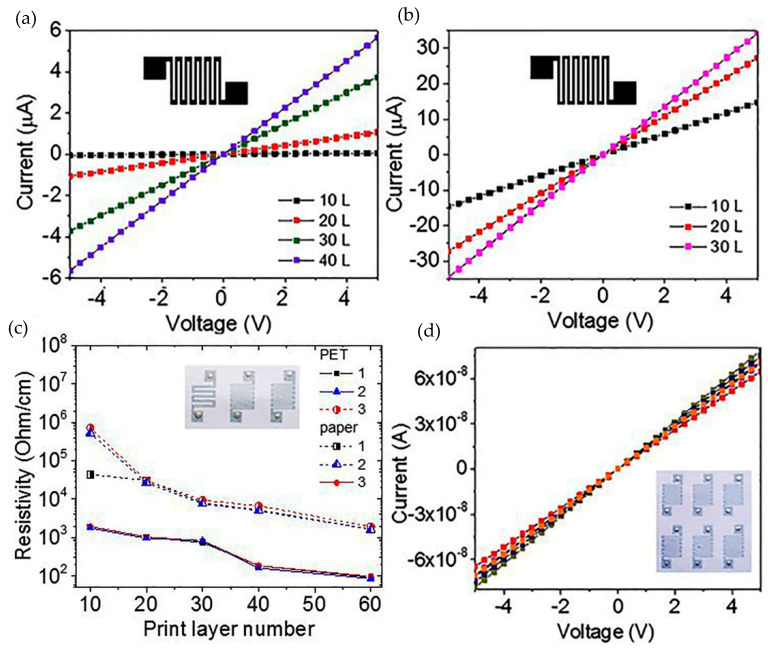
(**a**,**b**) Current-voltage characteristics for the structures with the different number of the printing layers (denoted as the parameter) on the different substrates: (**a**) the structure 3 on the paper and (**b**) the structure 2 on the polyvinyl terephthalate (PET). (**c**) Resistances of the different type structures on the different substrates depending on the number of the printing layers. The inserts in (**c**,**d**) give optical images of the three types of structures. The size of the structures 2 and 3, without the contacts, equal to 5 × 5 mm^2^. (**d**) Current–voltage characteristics for six similar structures from the insert (12 layers printed on the paper).

We printed using the composite ink made of the C-He suspension kept for 8 months. Here, to obtain a homogeneous composite suspension, we only applied a brief ultrasound and centrifuge treatment. As a result, we printed the resistive structures on two types of substrate (plain paper and polyvinyl terephthalate, PET). The insert in Figure 7c represents the structure photos: three structures are different in the strip parameters. Figure 7a,b show the current-voltage characteristics of the structures for the different numbers of printed layers. Figure 7c shows the total data on the resistance of the different type structures on the different substrates depending on the number of the printed layers. All other things being equal, the resistance of the layers on the PET is about 2–2.5 orders of magnitude lower than that on the paper. This difference is due to the large relief of the paper as compared to the PET. From the AFM measurement results, we estimate the thickness of the 40-layer printed layer as 350 nm. The layer resistances obtained for the 350–500 nm thickness of the printed structures were about 70–430 Ω/sq. These values are the minimum possible resistance values for the graphene layers created from a suspension. Figure 7d shows the current-voltage characteristics of 6 identical structures.

It is an important fact that printing of the films and practically transparent layer using the C-He suspension without PEDOT:PSS (five printing layers) on the paper results in obtaining the conductive track, several nanometers thick, with the resistance of 470 GΩ/sq, without transition to the composite films.

**Figure 8:**
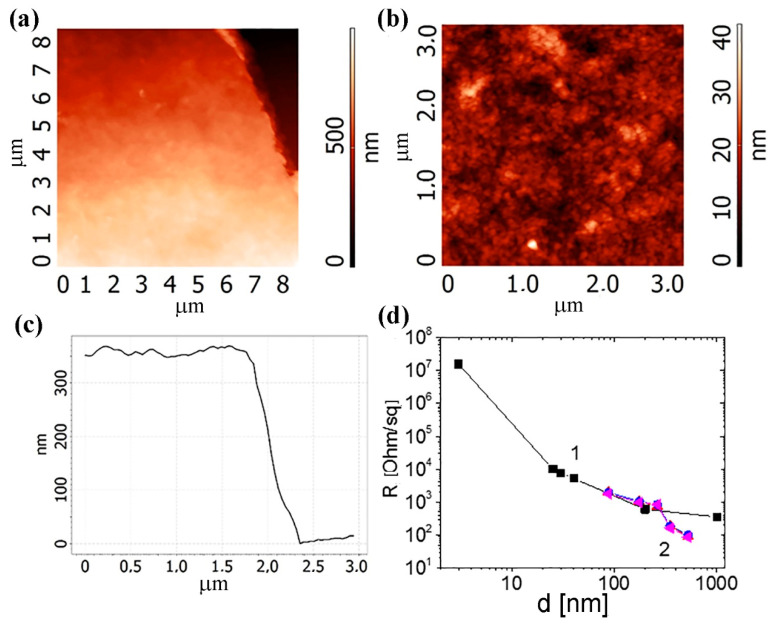
(**a**,**b**) AFM-images of 40 printed layers on the SiO_2_/Si substrate. (**c**) The thickness of the 40 printing layer of C-He: PEDOT:PSS (poly(3,4-ethylenedioxythiophene): polystyrene sulfonate) composite. (**d**) Dependences of the printed layer resistances from (1) the C-M suspension and (2) the C-He: PEDOT:PSS composite suspension.

Figure 8 shows the AFM images of 40 printed layers from the C-He: PEDOT:PSS composite suspension on the SiO_2_/Si substrate. Note that the layers are porous and straight. In general, mechanical testing of such layers, both on the paper and on the PET (bends of the printed structures) demonstrates the formation of the unified layer. Deformations did not lead to the printed film destruction. A comparison of the resistances of the films obtained from the C-M particles with the C-He-based composite films (Figure 8d) shows similar or noticeably lower values of the film resistances for C-He: PEDOT:PSS on the PET despite the relatively high resistance of PEDOT:PSS itself. It worth mentioning that Current-voltage (I-V) characteristics for C-M films with different thickness *d* are linear [14] similar to I-V of C-He shown in Figure 7.

**Figure 9:**
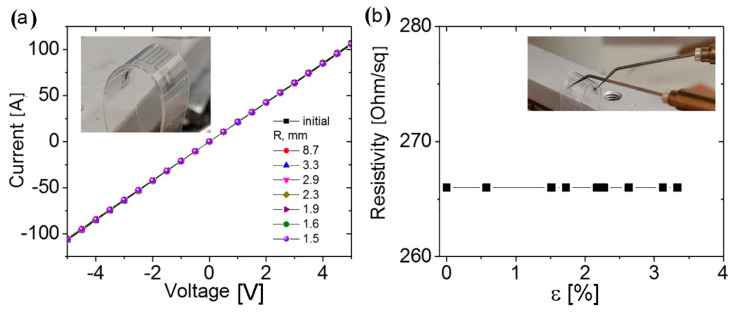
(**a**) Current-voltage characteristics of the structures from the graphene: PEDOT:PSS composite material (40 printed layers) measured at the different structure bend radius given as the parameter. (**b**) Structure resistance depending on the stretching deformation. Inserts are the photos of the bending structure during the measuring process.

Figure 9 shows the current-voltage characteristics of the structures made of the graphene: PEDOT:PSS composite material, measured at the different bending structure radii, and their resistance depending on the deformation. Note that up to the bend radius of 1.5 mm with respect to the strain of 3.3%, the characteristics and the resistance do not change.

For comparison, the films created from C-M suspension under similar tensile bending were demonstrated pronounced changes in the resistance (increase in resistance up to 30% for strain ~2%) and some scattering in the resistance values (10–15%) at the repeated measurements.

## 4. Discussion

To obtain graphene or multigraphene within a short period, various plasma generators are engaged [16,40,41,42]. Here, either substrate is used, or graphene is obtained in the free-standing state, that is, in a volume. In the first case, the substrate area and properties limit the efficiency of the method. In the second case, it is possible to continuously obtain the powder from the nanoparticles. The powder particles synthesized using a plasma jet always have the form of flakes; yet, the geometric parameters of these flakes can be controlled by changing the plasma-forming gas. In turn, the synthesized particle thickness will govern the future mechanical, structural, and electrical properties of the layers and the devices made of these particles. The fact is that only 1–2-layer flakes can combine into the unified film due to their flexibility and emergence of the sufficiently strong van der Waals interaction [14]. In the present study, we show that in the case of helium plasma, thin particles form (their thickness reaches 2 nm, the number of layers being 1–5). Using argon, about 10 nm thick particles are synthesized. This difference in properties may be engendered by the features of equilibrium plasma composition investigated here using quasi-one-dimensional modeling. It is shown that in the case of argon the environment encloses more radicals, electrons, and ions compared with the case of helium. This is the precondition to increase the number of condensed cycles and side reactions, favoring the increase in size and thickness of flakes and density of defects. This prediction of modeling agrees well with experimental data. As a result, the C-Ar flakes turned out to be non-conductive, in contrast to the C-He flakes. The absence of the C-Ar conductivity is most likely caused either by the formation of the amorphous particle or by the particle functionalization, turning, as a rule, graphene into a dielectric [43,44,45].

The bottom-up synthesis method implies that the nanostructures are synthesized by stacking atoms onto each other: this gives rise to the crystal planes; and the crystal planes further stack onto each other resulting in the nanomaterial synthesis. Despite its short history (compared to the other techniques), the plasma-based technologies have already achieved successes for the synthesis of the carbon-based materials [27,46,47]. In contrast to the other approaches (for instance, the CVD techniques), the plasma techniques do not rely on the use of a catalyst, surfactants, or other unnecessary substances. Moreover, plasma synthesis includes a promise for the facile, efficient, and mild modification of carbon materials. Among all kinds of approach, plasma-based synthesis is widely used due to its numerous advantages, such as highly distributed active species, reduced energy requirements, scalable production, and control of material parameters. But plasma synthesis might be accompanied by the formation of defective or amorphous graphene. Therefore, the main problem of this approach is the synthesis of the material with low structural defect concentration. As follows from the Raman data and the electrical properties, this problem was successfully solved in the case of using the helium plasma.

Analysis of electrical properties and the search for electronic applications are very poorly represented in the available literature. The exception is an electrochemical application in fuel cells [46], lithium-ion batteries [47], and supercondensators [30]. Electrical properties are a key issue for a wide spectrum of applications. Generally, it should be mentioned that the plasma synthesized materials, including graphene, are characterized by the standpoint of their structural properties, and chemical content of flakes [30,48,49,50,51]. Here, we study the plasma jet-assisted synthesized graphene materials aimed at the development of electronic devices fabricated by printing techniques on a flexible substrate, providing pervasive, light-weight, and cost-effective devices. As shown above, the composite films based on the C-He and the PEDOT:PSS graphene particles have fairly low resistance and form even porous layers in printing. Accounting for the relatively high carrier mobility in these C-He films and their translucency, despite the relatively large thickness, they are extremely promising for creating the conductive layers for a variety of sensors, transistors, and other electronic devices. The porosity of the printed layers makes them particularly promising for the resistive sensors of gases, humidity, etc.

The study of their structural flexibility shows both good adhesion to the composite film, to PET, and the stable layer properties under the bending up to 1.5 mm (tensile strain ~3%), thus providing the prospects of such a material and structures for flexible electronics. The flexibility of films created from C-M suspension was studied where changes in the resistance and some scattering in the resistance values under repeated measurements (within 30% for strain ~2%) (see also Ref. [52]) were demonstrated. It is important to mention here that these films consist of a large number of flakes, and the basal planes of flakes are located not only horizontally on the substrate, but some of the flakes also have different positions. This effect is expected for C-M films due to the relatively large thickness of flakes. In this case, as a result of the film bending, the flakes are subjected to different types of deformation (symmetric and asymmetrical stretching, shear, and non-uniform deformations). According to the theoretical study, the asymmetrical stretching, shear, and non-uniform deformations lead to the strongest bending effects connected with the bandgap opening [53,54,55]. The resulting bending effect was found to vary under repeated measurements on such a mesoscopic system. Even small changes in the tested structure fixation in the holder result in a variable reaction to the bending. In contrast, the composite C-He:PEDOT:PSS films show the excellent reproducibility in their resistance values under tensive strain due to bending. The best stability of the C-He:PEDOT:PSS films during deformation is based on a good structure of films due to the thin graphene flakes, resulting in good interaction between flakes.

To the best of our knowledge, this work paves the path to use of these kind graphene flakes in printed flexible sensors, logics, and other devices aimed at use in the continuously expanding Internet of Things ecosystem.

## 5. Conclusions

We show that graphene flakes synthesized in helium plasma with the propane-butane mixture addition can be used to obtain a water-based graphene suspension or a composite suspension with PEDOT:PSS to create structures using the 2D printing technologies. The low thickness (from a monolayer to 2 nm) and the high conductivity of the layers obtained from such particles is one of the important and attractive parameters of such particles. For a bottom-up synthesis approach, the formation of a material with that low density of defects is not an easy task. On the contrary, the particles synthesized in argon plasma are thicker, more defective, and non-conductive.

One-dimensional flow modeling shows that in the case of argon the environment encloses more radicals, electrons, and ions compared with helium. This is the precondition to increase the number of condensed cycles and side reactions, favoring the increase in size and thickness of flakes and defect density. In contrast, helium plasma leads to the creation of graphene flakes C-He with more promising application properties.

Some test structures were fabricated from C-He suspension with the use of 2D printed technologies. The predominance of monolayer graphene flakes created in helium plasma provides the unique stability of the water-based graphene inks (at least 8 months). The composite graphene: PEDOT:PSS films have a smooth surface and a uniform structure. The layer resistance of the relatively thick composite layers obtained by droplets equals 600–800 Ω/sq for the submicron thickness film and to 1.4–2.6 kΩ/sq for a thin film (about 200 nm). The resistance of the ~80–500 nm thick layers created by the 2D printing on the PET was equal to 70–2000 Ω/sq: significantly lower than that of the graphene flakes created by graphite exfoliation. In general, good structural quality, low layer resistance, sufficiently high carrier mobility, and good mechanical strength combined with the possibility to obtain a large amount of the graphene powder, and the possibility to control the parameters of the synthesized particles, makes this material promising, above all, for resistive sensors. Our results contribute to the direct integration of the graphene structures into the 2D printing technology on flexible substrates for nanoelectronics, sensors, biomedical, and optoelectronic components and nanodevices.

## Figures and Tables

**Table 1:** Parameters of the films obtained from the different types of suspensions. The film thickness was about a micrometer. *R*_S_ is the layer resistance, *R*_SC_ is the layer resistance of the composite film, μ is the carrier mobility of the composite film.

Flake Type	Thickness, nm	Flake Size, nm	Film *R*_S_, kΩ/sq	Composite Film *R*_S_, kΩ/sq	μ cm^2^/*V*s
C-Ar	2–10	50–800	1500	4–0	<1
C-He	0.4–2	50–200	4.5	0.4–0.8	6–90
C-M	4–10	1–5 μm	3–5	1–2	<1
